# Analysis of definitive chemo-radiotherapy for esophageal cancer with supra-clavicular node metastasis based on CT in a single institutional retrospective study: a propensity score matching analysis

**DOI:** 10.1186/s13014-018-1145-4

**Published:** 2018-10-16

**Authors:** Hong-Yao Xu, Sheng-Xi Wu, He-San Luo, Chu-Yun Chen, Lian-Xing Lin, He-Cheng Huang

**Affiliations:** Department of Radiotherapy, Shantou Central Hospital (The Affiliated Shantou Hospital of Sun Yat-sen University), Shantou, 515031 China

**Keywords:** Supra-clavicular lymph node, Esophageal cancer, Definitive chemo-radiotherapy

## Abstract

**Background:**

The prognostic value of supra-clavicular lymph node (SCLN) metastases in esophageal cancer (EC) is still not clear.

**Method:**

From January 2009 to December 2015, a survival analysis was performed to retrospectively identify the prognostic value of SCLN metastasis on survival on 751 patients with EC treated with definitive chemo-radiotherapy (dCRT).

**Results:**

The median follow-up duration for living patients was 56.6 months. The median overall survival (OS) for all patients was 16.6 months. Patients with SCLN metastasis had a much poorer prognosis for OS (*χ*^*2*^ *=* 17.342, *P* < 0.001), distant metastasis-free survival (DMFS) (*χ*^*2*^ *=* 24.793, *P <* 0.001) and progression-free survival (PFS) (*χ*^*2*^ *=* 25.802, *P <* 0.001) than those without SCLN metastasis. The same results were found after propensity score matching. Nonetheless, the prognosis of patients with cervical or upper thoracic EC metastasis in SCLN was better than those of patients with middle or lower thoracic EC metastasis in SCLN for OS (*χ*^*2*^ *=* 4.516, *P =* 0.038), DMFS (*χ*^*2*^ *=* 8.326, *P* = 0.004) and PFS (*χ*^*2*^ *=* 6.255, *P =* 0.012). Univariate analysis showed that gender, middle or lower thoracic EC with SCLN metastasis, tumor length, tumor diameter, concurrent chemo-radiotherapy (CCR) and number of lymph nodes were prognostic factors for PFS. Gender, age, middle or lower thoracic EC with SCLN metastasis, tumor diameter, tumor length, and number of lymph nodes were prognostic factors for DMFS. According to the multivariate analysis, only middle or lower thoracic EC with SCLN metastasis and number of lymph nodes were independent prognostic factors for DMFS and PFS.

**Conclusion:**

For patients with cervical or upper thoracic EC, metastasis in SCLN should be considered to be regional lymph nodes and treated with curative intent if the total number of lymph nodes is limited. However, for patients with middle or lower thoracic EC, metastasis should be considered to be a higher level N stage or M1 stage, and it is thus necessary to provide consolidation chemotherapy after dCRT.

## Background

Esophageal cancer (EC) greatly threatens human health in China [1], and supra-clavicular lymph node (SCLN) metastasis accounts for approximately 8–20% of patients with EC, which have a much poorer prognosis [2–8]. In the 7th edition of TNM staging, SCLN metastasis is defined as distant metastasis (M) and thus prognostically unfavorable [9]. However, in the 8th edition of TNM staging, it is defined as regional lymph nodes [10]. which is based on many studies performed on patients treated with surgery. In patients treated with dCRT, N stage disease is considered a prognostic factor [6–8]. However, whether SCLN metastasis should be considered as N stage or M1 stage is still not clear. Additionally, different locations of EC with SCLN may not have the same prognosis, which is rarely analyzed separately. The aim of the current study was to identify the prognostic value of SCLN for EC treated with dCRT.

## Methods

From January 2009 to December 2015, a total of 751 untreated patients with EC treated with dCRT at Shantou Central Hospital (the Affiliated Shantou Hospital of Sun Yat-sen University) were retrospectively reviewed. Data were retrieved in May 2018, ensuring a minimum potential follow-up duration of 24 months. All patients had histologically confirmed EC and no distant metastasis or combined with another tumor. Clinical stage was performed according to the 8th edition of TNM staging using barium esophagography, a computed tomography (CT) scan and electronic and ultrasound gastroscopy. PET/CT was not routinely carried out.

### Statistical analysis

The Kaplan–Meier method was used to estimate survival outcomes, and differences in survival were compared using the log-rank test. A progression-free event was defined as the first documented radiographic evidence of progressive disease or death from any cause. Cox regression was used to evaluate independent prognostic factors associated with OS and PFS. *P* < 0.05 indicated a significant deference. The propensity score matching (PSM) analysis (including variables such as age, sex, histology, tumor length, tumor diameter, tumor location, T stage, nodal status, stage, and chemotherapy) was performed using a one-to-one nearest neighbor method. All *P* values and 95% confidence intervals (95% CIs) are two-sided. Statistical analyses were performed using the Statistic Package for the Social Sciences (SPSS, version 22.0).

### Radiotherapy

All patients had a CT or PET/CT scan; the treatment position was supine with the arms raised above the head. For patients with a proximal tumor, head and neck shoulder film or a vacuum pad was used and the arms were placed next to the body. External irradiation was performed with a 6 MV X-ray linear accelerator. A total of 709 patients were treated with three-dimensional conformal radiotherapy. Forty-two patients were treated with intensity-modulated radiation. The median radiotherapy dose was 64 Gy (46~ 70 Gy). The target areas were evaluated by two radiologists and any discrepancy was resolved by discussion. The gross tumor volume (GTV) includes imaging positive lesions; GTV-N includes the clinical diagnosis of positive lymph nodes (SCLN > 5 mm [11], mediastinal tracheal fork above the lymph node diameter > 5 mm; tracheal bifurcation below the lymph node diameter > 10 mm [12]). The clinical target volume (CTV) was contoured based on the GTV and GTV-N with the external expansion of 3 cm (up and down direction) and outside the expansion of 0.5 cm (before and after; left and right direction). The CTV of the upper thoracic EC includes the bilateral supra-clavicular region. The PTV was calculated on the basis of the CTV outside the expansion of 1 cm (up and down direction) and 0.5 cm (before and after; left and right direction). Radiation was delivered with the following normal tissue constraints: < 30% volume of the lungs receiving 20 Gy; < 50% volume of the heart receiving 45 Gy; and < 10% volume of the spinal cord receiving 50 Gy (Dmax < 50 Gy). CCR consisted of weekly concurrent docetaxel, cisplatin or nedaplatin (25 mg/m^2^) targeted at five to six courses in total. The CCT regimen consisted of two to four cycles of platinum-based chemotherapy (20–25 mg/m^2^, days 1–3) with 5-FU (750 mg/m^2^, days 1–4) or docetaxel (75 mg/m^2^, day 1) every 28 days.

## Results

### Patients

Between January 2009 and December 2015, a total of 751 patients with EC who received dCRT were identified and their medical records were reviewed. Of the 751 patients, 155 (20.6%) had SCLN metastasis, 45 (29%) of which had cervical or upper thoracic EC and 110 (71%) of which had middle or lower thoracic EC. As seen in Table 1, in the SCLN-positive group, more patients presented with a much higher disease stage, both for T stage and N stage. However, after PSM, the patient characteristics between the two groups included 140 patients; each group was well balanced.

**Table 1:** patient characteristics(*n* = 751)

Characteristic		SCLN status			SCLN status(after matching)		
		Negative (*n* = 596)	Positive (*n* = 155)	*P* value	Negative (*n* = 140)	Positive (n = 140)	*P* value
Age (years)	≤65 years	306(51.3%)	99(63.9%)	0.063	90(64.3%)	85(60.7%)	0.537
	> 65 years	290(48.7%)	56(36.1%)		50(35.7%)	55(39.3%)	
Sex	Male	432(72.5%)	121(78.1%)	0.348	104(74.3%)	108(77.1%)	0.577
	Female	164(27.5%)	34(21.9%)		36(25.7%)	32(22.9%)	
Histology	SCC	567(95.1%)	143(92.3%)	0.390	134(95.7%)	130(92.9%)	0.352
	Others	29(4.9%)	12(7.7%)		6(4.3%)	10(7.1%)	
Tumor length	≤5 cm	207(34.7%)	32(20.6%)	0.027	30(21.4%)	30(21.4%)	1.000
	> 5 cm	389(65.3%)	123(79.4%)		110(78.6%)	110(78.6%)	
Tumor diameter	≤3 cm	210(35.2%)	34(21.9%)	0.042	37(26.4%)	30(21.4%)	0.327
	> 3 cm	386(64.8%)	121(78.1%)		103(73.6%)	110(78.6%)	
T stage	T1/T2	190(31.9%)	29(18.7%)	0.002	27(19.3%)	27(19.3%)	0.175
	T3	174(29.2%)	27(17.4%)		39(27.9%)	24(17.1%)	
	T4	232(38.9%)	99(63.9%)		74(52.9%)	89(63.6%)	
N stage	N0	158(26.5%)	0(0%)	0.000	17(12.1%)	0(0.0%)	0.180
	N1	312(52.3%)	33(21.3%)		24(17.1%)	33(23.6%)	
	N2	111(18.6%)	92(59.4%)		84(60.0%)	88(62.9%)	
	N3	15(2.5%)	30(19.4%)		15(10.7%)	19(13.6%)	
Chemotherapy	Yes	384(64.4%)	129(83.2%)	0.002	32(22.9%)	26(18.6%)	0.376
	No	212(35.6%)	26(16.8%)		108(77.1%)	114(81.4%)	
Tumor location	Cervical	37(6.2%)	9(5.8%)	0.869	6(4.3%)	9(6.4%)	0.792
	Upper	121(20.3%)	36(23.2%)		35(25.0%)	32(22.9%)	
	Middle	332(55.7%)	88(56.8%)		78(55.7%)	80(57.1%)	
	Lower	106(17.8%)	22(14.2%)		21(15.0%)	19(13.5%)	
number of LN	0	160(26.8%)	0(0.0%)	0.000	17(12.1%)	0(0.0%)	0.137
	1–2	310(52.0%)	34(21.9%)		24(17.1%)	34(24.3%)	
	> 3	126(21.1%)	121(78.1%)		99(70.7%)	106(75.7%)	

### Survival

From the beginning of treatment until May 31, 2018, the median follow-up duration for living patients was 56.6 months (range 25.2–112.5 months). Only 2 cases were lost to follow-up and were defined as censored cases.

**Fig. 1:**
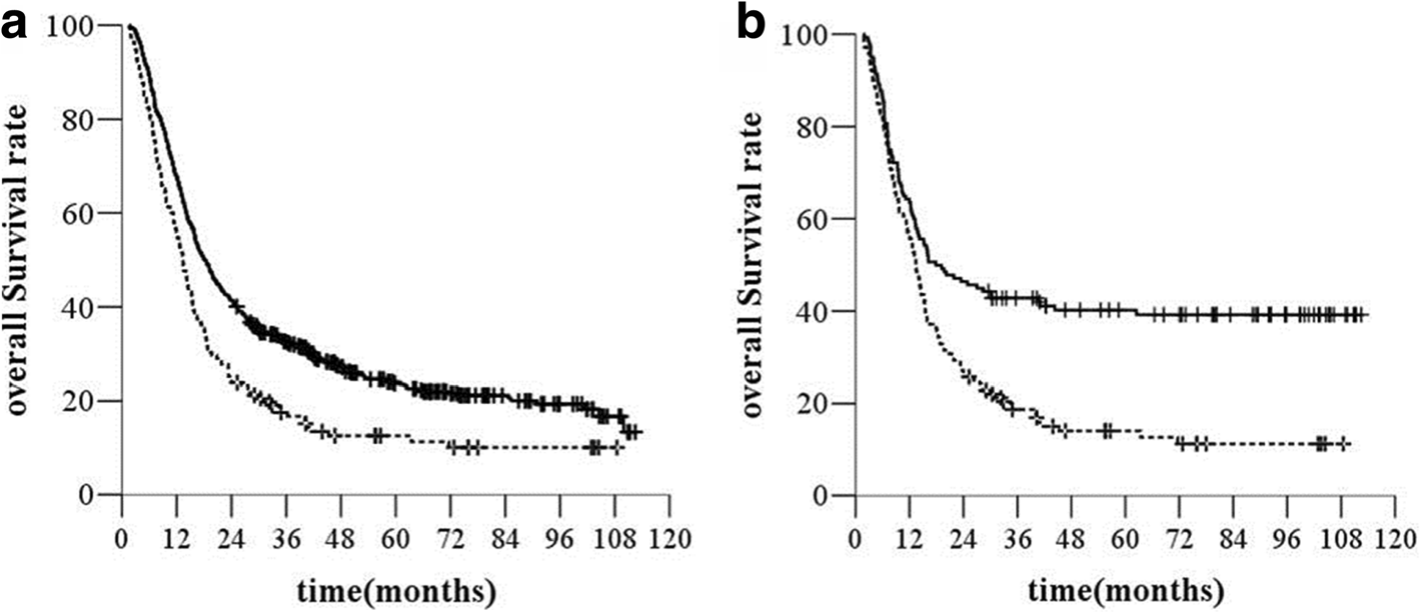
Overall survival (OS) before (**a**) and after (**b**) propensity score matching. legend: —without SCLN, −--with SCLN

The median OS time in the SCLN-negative group was 18.1 months (95% *CI*, 16.1–20.1 months) and that in the SCLN-positive group was 13.4 months (95% *CI*, 11.7–15.1 months) (*χ*^*2*^ *=* 17.342, *P* < 0.001, *HR* = 1.532, 95% *CI*, 1.263–1.858, Fig. 1a). The 1-, 3- and 5-year OS rates were 56.1%, 17.6%, and 12.6%, respectively, in the SCLN-positive group and 67.8%, 32.6%, and 23.8%, respectively, in the SCLN-negative group.

**Fig. 2:**
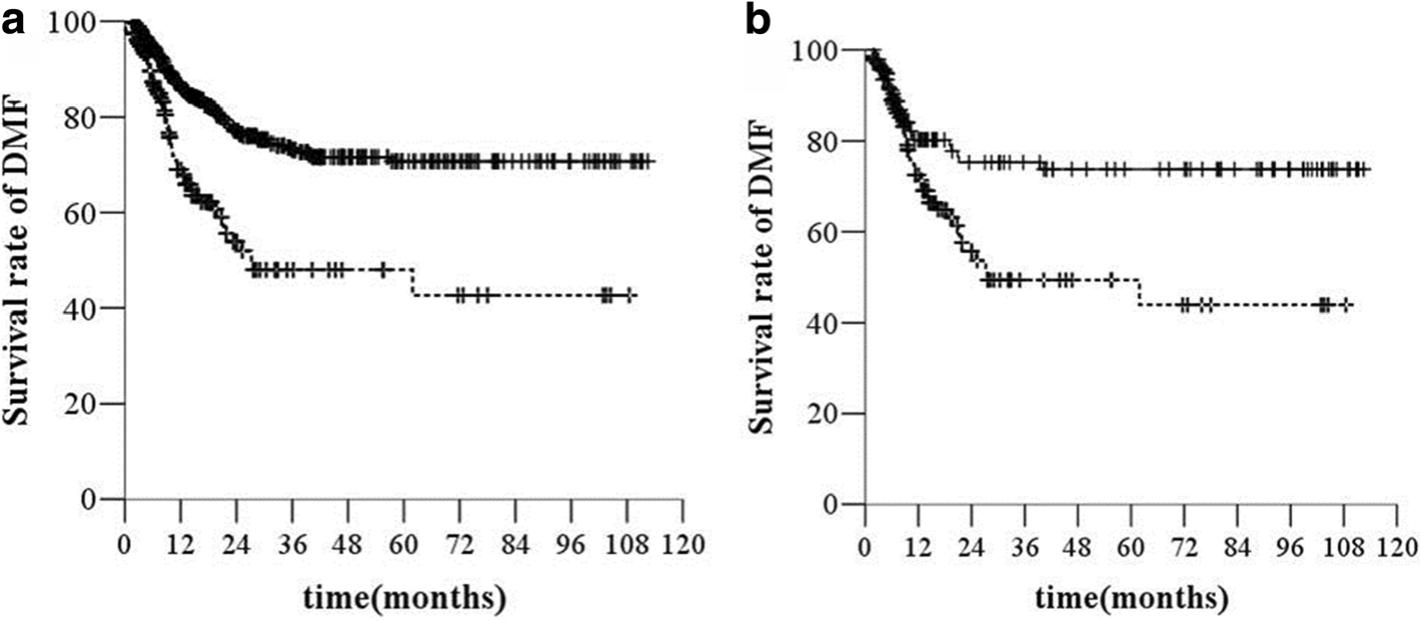
DMFS before (**a**) and after (**b**) propensity score matching. Legend: —without SCLN, −--with SCLN

The SCLN-positive group had a higher rate of distant metastases than the group without SCLN metastasis (53 [34.2%] of 155 versus 117 [19.6%] of 596) (*χ*^*2*^ *=* 24.793, *P <* 0.001, *HR* = 2.421, 95% *CI*, 1.747–3.356, Fig. 2a).

**Fig. 3:**
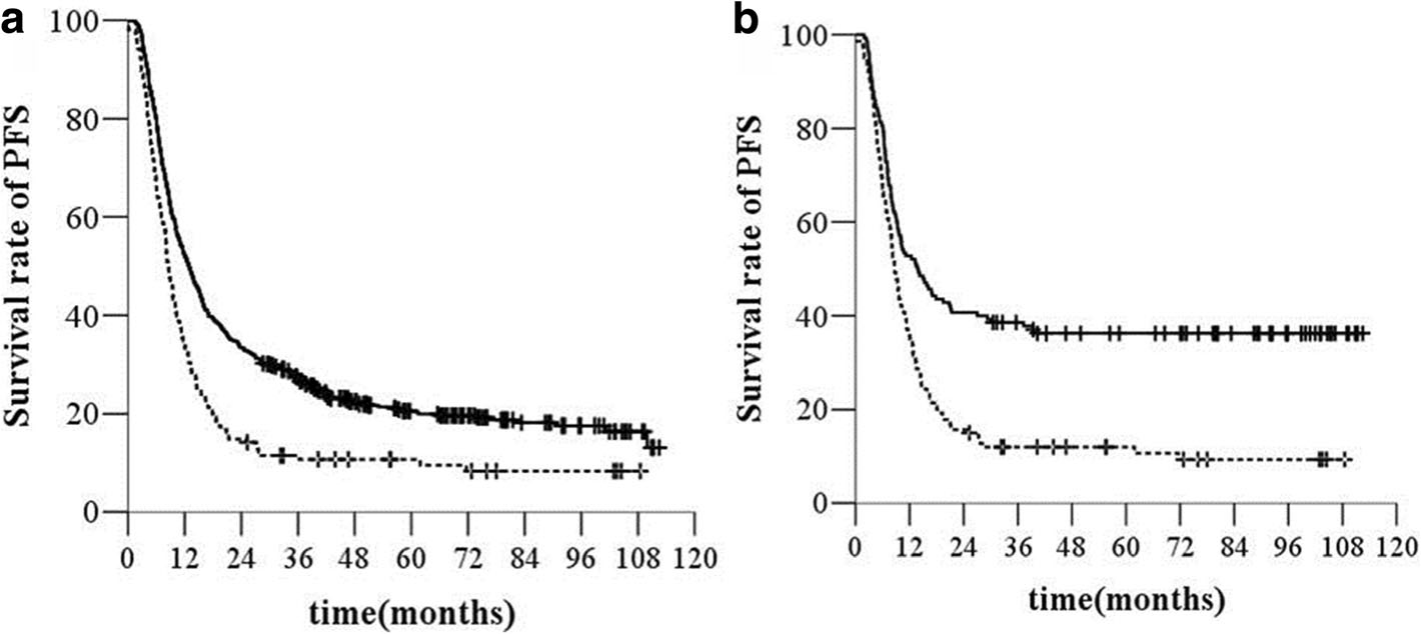
PFS before (**a**) and after (**b**) propensity score matching. Legend: —without SCLN, −--with SCLN

The 1-, 3-, and 5- year PFS rates were 65.4%, 29.6% and 21.5%, respectively, in the SCLN-negative group and 48.5%, 24.1% and 18.5%, respectively, in the SCLN-positive group. The median PFS time in the SCLN-negative group was 12.9 months (95% *CI*, 1 1.3–14.5 months) and that in the SCLN-positive group was 8.6 months (95% *CI*, 7.5–9.6 months) (*χ*^*2*^ *=* 25.802, *P <* 0.001, *HR* = 1.627, 95% *CI*, 1.346–1.968, Fig. 3a).

After matching (140 patients in each treatment group), the median OS was 17.9 months (95% *CI*, 8.01–27.7) in the SCLN-negative group and 13.4 months (95% *CI*, 11.8–15.0) in the SCLN-positive group (*χ*^*2*^ *=* 17.14, *P* < 0.000, *HR* = 1.798, 95% *CI*, 1.357–2.384, Fig. 1b). The SCLN-positive group had a higher rate of distant metastasis than the SCLN-negative group (45 [32.1%] of 140 versus 29 [20.7%] of 140) (*χ*^*2*^ *=* 8.446, *P =* 0.004, *HR* = 1.986, 95% *CI*, 1.239–3.182, Fig. 2b). The median PFS time was 13.7 months (95% *CI*, 8.2–19.3 months) in the SCLN-negative group and 8.6 months (95% *CI*, 7.4–9.8 months) in the SCLN-positive group (*χ*^*2*^ *=* 22.132, *P <* 0.000, *HR* = 1.915, 95% *CI*, 1.454–2.523, Fig. 3b).

### Subgroup analysis of SCLN metastasis

All patients with SCLN metastasis were divided into two groups to analyze the relationship between SCLN metastasis and location of the primary tumor.

**Fig. 4:**
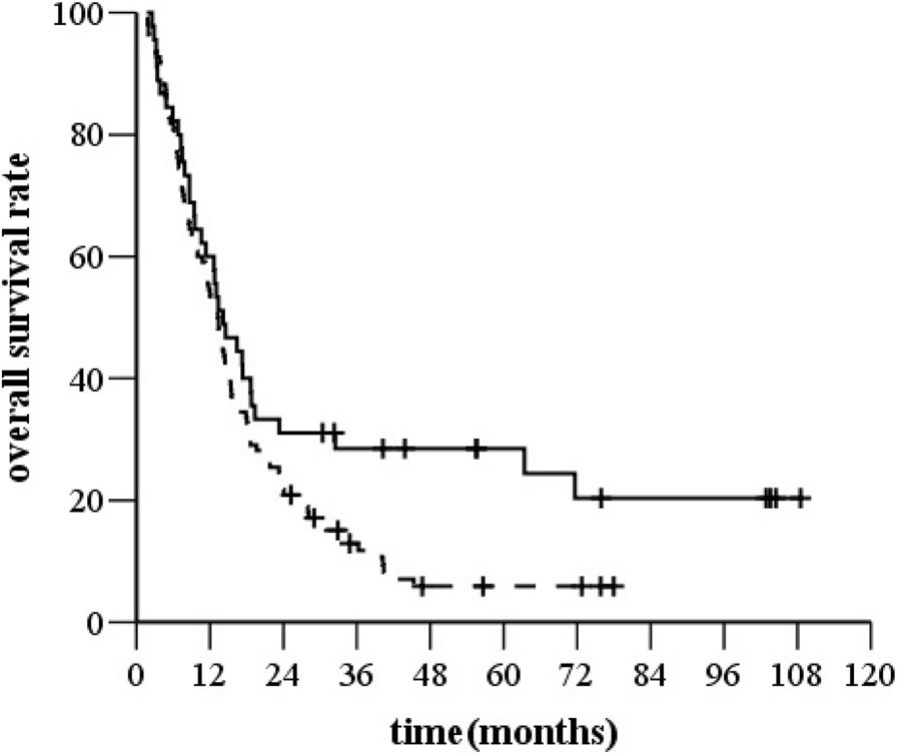
Overall survival (OS) for patients with different locations of the primary tumor. Legend: —cervical or upper thoracic EC with SCLN, −-- middle or lower thoracic EC with SCLN

The 1-, 3- and 5-year OS rates in patients with cervical or upper thoracic EC metastasis in SCLN were 60%, 28.5%, and 24.4%, respectively, and those in patients with middle or lower thoracic EC metastasis in SCLN were 54.5%, 12.9%, and 5.9%, respectively, (*χ*^*2*^ *=* 4.516, *P =* 0.038, *HR* = 1.512, 95% *CI*, 1.020–2.241, Fig. 4).

**Fig. 5:**
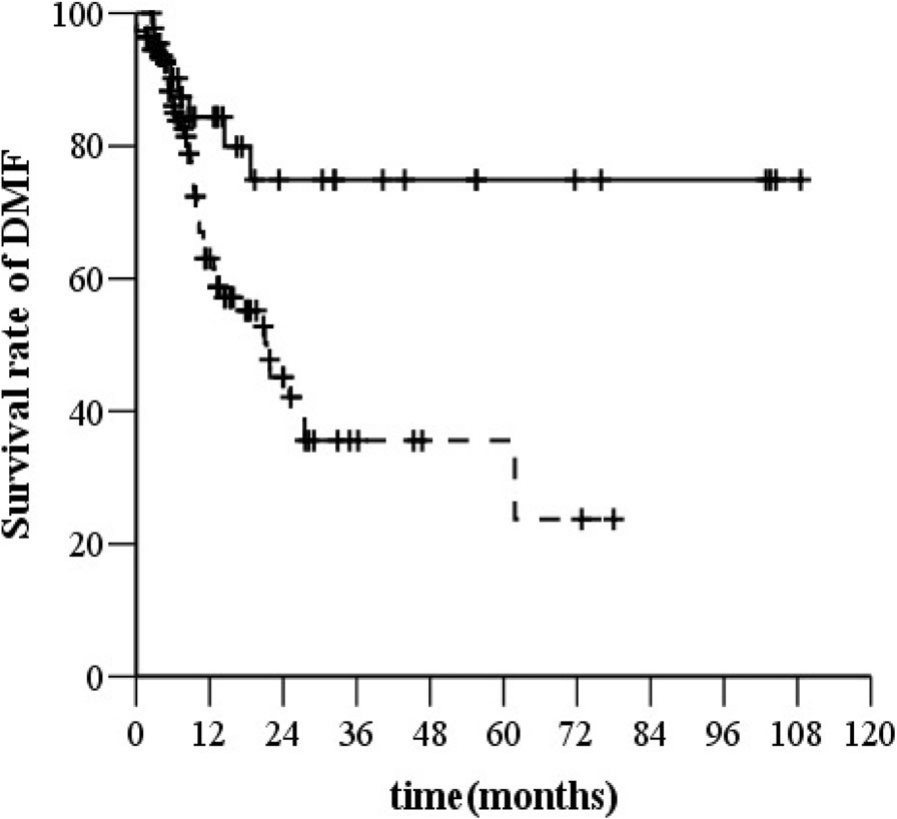
Distant metastasis-free survival (DMFS) for patients with different locations of the primary tumor. Legend: —cervical or upper thoracic EC with SCLN, −-- middle or lower thoracic EC with SCLN

Patients with middle or lower thoracic EC with SCLN metastasis had a higher rate of distant metastases than those with cervical or upper thoracic EC with SCLN metastasis (45 [40.9%] of 110 versus 8 [17.8%] of 45, *χ*^*2*^ *=* 8.326, *P* = 0.004, Fig. 5).

**Fig. 6:**
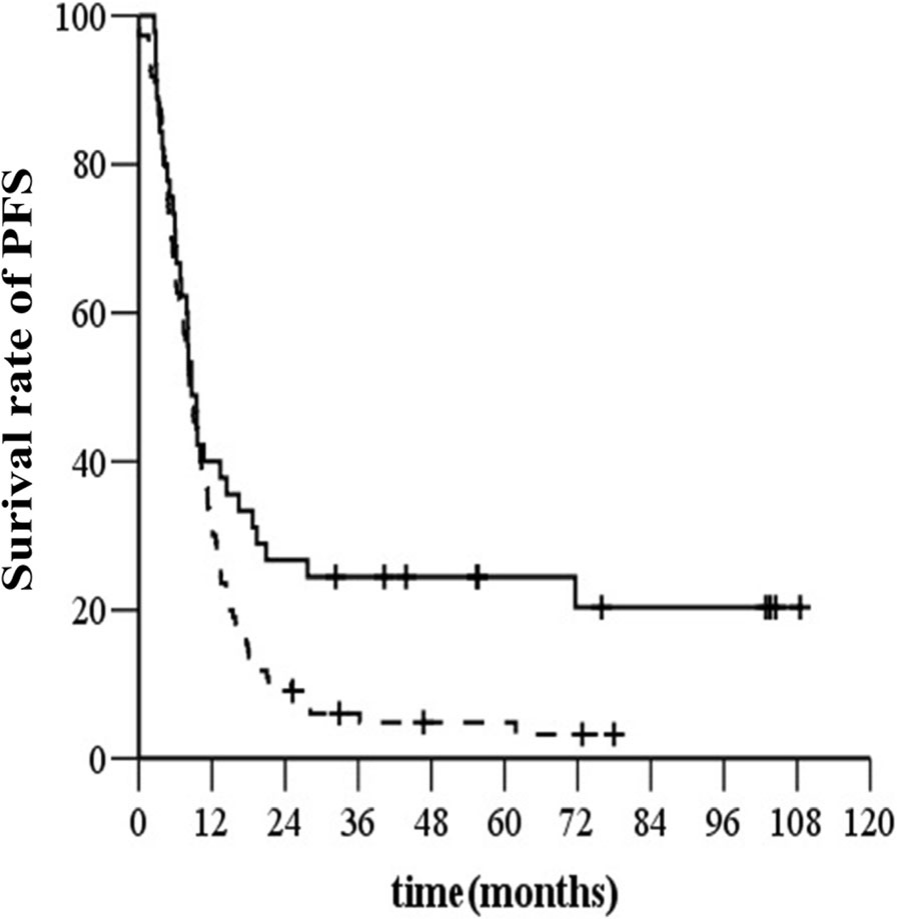
Progression-free survival (PFS) for patients with different locations of the primary tumor. Legend: —cervical or upper thoracic EC with SCLN, −-- middle or lower thoracic EC with SCLN

The 1-, 3- and 5-year PFS rates in patients with cervical or upper thoracic EC metastasis in SCLN were 40%, 24.4%, and 20.4%, respectively, and those in patients with middle or lower thoracic EC metastasis in SCLN were 31.8%, 6.1%, and 3.2%, respectively (*χ*^*2*^ *=* 6.255, *P =* 0.012, *HR* = 1.616, 95% *CI*, 1.094–2.386, Fig. 6).

### Risk factors for survival

Univariate analysis showed that gender, middle or lower thoracic EC with SCLN metastasis, tumor length, tumor diameter, CCR and number of lymph nodes were prognostic factors for PFS. Gender, age, middle or lower thoracic EC with SCLN metastasis, tumor length, tumor diameter, and number of lymph nodes were prognostic factors for DMFS. However, cervical or upper thoracic EC with SCLN metastasis was not statistically significant for DMFS and PFS (Table 2).

**Table 2:** Univariae analysis of prognostic factors for patients with esophageal cancer

Variable	Harzard ratos for PFS		Harzard ratos for DMFS	
	*P* value	HR(95%CI)	*P*	HR(95%CI)
gender	0.000	0.708(0.587–0.853)	0.005	0.586(0.403–0.854)
age	0.972	1.003(0.856–1.175)	0.001	0.595(0.436–0.813)
Middle or lower with SCLN	0.000	1.897(1.534–2.348)	0.000	3.116(2.207–4.398)
upper or cervical with SCLN	0.841	1.036(0.736–1.457)	0.709	0.874(0.430–1.777)
diameter	0.000	1.725(1.489–1.998)	0.000	1.786(1.351–2.362)
length	0.000	1.631(1.406–1.891)	0.000	1.797(1.355–2.383)
CCR	0.018	0.824(0.703–0.967)	0.139	1.268(0.926–1.737)
CCT	0.283	0.912(0.770–1.079)	0.055	1.345(0.993–1.822)
Number of lymph node	0.000	1.387(1.128–1.492)	0.000	1.682(1.459–1.938)

All factors influencing prognosis were analyzed by Cox multivariate analysis. Only middle or lower thoracic EC with SCLN metastasis and number of lymph nodes were prognostic factors for DMFS and PFS (Table 3).

**Table 3:** Multivariate analysis of prognostic factors for patients with esophageal cancer

Variable	Harzard ratos for PFS		Harzard ratos for DMFS	
	*P* value	HR(95%CI)	*P*	HR(95%CI)
gender	0.004	0.756(0.625–0.914)	0.091	0.720(0.491–1.054)
age	–		0.015	0.676(0.493–0.927)
Middle and lower with SCLN	0.025	1.303(1.033–1.644)	0.002	1.819(1.242–2.664)
diameter	0.000	1.407(1.169–1.693)	0.161	1.281(0.906–1.810)
length	0.496	1.068(0.884–1.290)	0.931	1.016(0.713–1.447)
CCR	0.000	0.706(0.601–0.830)	–	
Number of lymph node	0.000	1.259(1.157–1.370)	0.000	1.426(1.207–1.685)

## Discussion

EC is a common malignant tumor in China, especially in the Chaoshan area. Most ECs have local advanced lesions and are thus excluded from curative surgery. According to the 8th edition of TNM staging, SCLNs are defined as lower cervical paratracheal nodes (1 L and 1R) [10]. It is recommended that SCLNs be considered as regional lymph nodes and treated with curative intent if the total number of involved lymph nodes is limited [6–8]. However, common sense suggests distal lymph node metastasis could cause more harm than proximal lymph node metastasis. According to this hypothesis, the impact of SCLN metastasis on long-term survival may be more prominent in patients with middle or lower thoracic EC. Jeene et al. showed that SCLN disease is not an independent prognostic factor for survival of EC patients treated with definitive chemoradiation [6]. As indicated by many studies, the outcomes of ESCC are not simply impacted by SCLN status, but are primarily determined by the number of involved nodes, including SCLNs [13–16]; however, only a few cases were analyzed and the SCLNs were not separated from the location of the primary tumor. A recent study reported that among patients with SCLN metastasis, the 5-year survival rate was 42.3%, 40.5%, and 30.0% for upper, middle, and lower EC, respectively [17]. These results suggest that patients with SCLN with middle or lower thoracic EC may have a worse prognosis. We can see the statistical significance when the SCLNs were separated from the primary tumor. All of the studies mentioned above reveal a trend that middle or lower thoracic EC with SCLN metastasis has a worse prognosis than cervical or upper EC with SCLN metastasis.

According to our results, SCLN had a much poorer prognosis and the same results were observed after PSM. However, significant differences in the prognosis of SCLN were observed by subgroup analysis. Middle or lower thoracic EC with SCLN metastasis is likely an independent prognostic factor for PFS and DMFS; this result is inconsistent with the most recent 7th edition of TNM staging. However, consistent with the 8th edition of TNM staging, the number of lymph nodes was prognostically unfavorable, which has also been reported by several recent studies [6–8].

Radiotherapists are eager to have more suitable non-surgical TNM staging for patients with EC rather than using the 8th edition of TNM staging, which is based on surgery. Our results complement the non-surgical N staging of EC.

This study has some limitations. It is retrospective and data were obtained from a single institution. Assessment of SCLN was determined by a CT scan and tumor staging was based on radiological examination. Finally, additional mechanistic and/or molecular results are needed to support our hypothesis.

## Conclusion

For upper or cervical EC, SCLN metastasis is still based on regional lymph node metastasis; however, for middle or lower thoracic EC, SCLN metastasis should be considered as distant lymph node metastasis and thus it is necessary to provide adjuvant treatment after dCRT.

## Abbreviations

CCR: concurrent chemo-radiotherapy
CT: computed tomography
dCRT: definitive chemo-radiotherapy
DMFS: distant metastasis-free survival
EC: esophageal cancer
OS: overall survival
PFS: progression-free survival
PSM: propensity score matching
SCLN: supra-clavicular lymph node
